# Synthetic Injectable Biomaterials for Alveolar Bone Regeneration in Animal and Human Studies

**DOI:** 10.3390/ma14112858

**Published:** 2021-05-26

**Authors:** Matej Tomas, Marija Čandrlić, Martina Juzbašić, Zrinka Ivanišević, Nikola Matijević, Aleksandar Včev, Olga Cvijanović Peloza, Marko Matijević, Željka Perić Kačarević

**Affiliations:** 1 Department of Dental Medicine, Faculty of Dental Medicine and Health Osijek, J.J. Strossmayer University of Osijek, 31 000 Osijek, Croatia; matej.tomas@fdmz.hr (M.T.); marija.candrlic@fdmz.hr (M.Č.); martina.juzbasic@fdmz.hr (M.J.); zrinkaivan@gmail.com (Z.I.); nm.matijevic@gmail.com (N.M.); 2 Interdisciplinary University Study of Molecular Biosciences, J.J. Strossmayer University of Osijek, 31 000 Osijek, Croatia; 3 Faculty of Medicine Osijek, J.J. Strossmayer University of Osijek, 31 000 Osijek, Croatia; 4 Department of Pathophysiology, Physiology and Immunology, Faculty of Dental Medicine and Health Osijek, J.J. Strossmayer University of Osijek, 31 000 Osijek, Croatia; avcev@fdmz.hr; 5 Department of Anatomy, Medical Faculty of the University of Rijeka, 51 000 Rijeka, Croatia; olga.cvijanovic@uniri.hr; 6 Department of Anatomy, Histology, Embriology, Pathology Anatomy and Pathology Histology, Faculty of Dental Medicine and Health Osijek, J.J. Strossmayer University of Osijek, 31 000 Osijek, Croatia

**Keywords:** injectable synthetic bone graft, alloplastic biomaterials, bone regeneration

## Abstract

After tooth extraction, the alveolar ridge undergoes dimensional changes. Different bone regeneration biomaterials are used to reduce bone loss. The aim of this article was to systematically review the literature on the effect of injectable synthetic biomaterials and their advantages and disadvantages for new bone formation in the maxilla and mandible in animals and humans. A literature search was conducted in November 2020 via MEDLINE PubMed, Cochrane, and Embase. Of the 501 records screened, abstract analysis was performed on 49 articles, resulting in 21 studies that met the inclusion criteria. Animal studies have shown heterogeneity in terms of animal models, follow-up time, composition of the injectable biomaterial, and different outcome variables such as bone–implant contact, newly formed bone, and peri-implant bone density. Heterogeneity has also been demonstrated by human studies. The following outcomes were observed: newly formed bone, connective tissue, residual injectable bone graft substitute, radiographic density, residual bone height, and different follow-up periods. Further studies, especially in humans, based on the histological and biomechanical properties of the injectable delivery form, are needed to draw more concrete conclusions that will contribute to a better understanding of the benefits of this type of biomaterials and their role in bone regeneration.

## 1. Introduction

**Figure 1:**
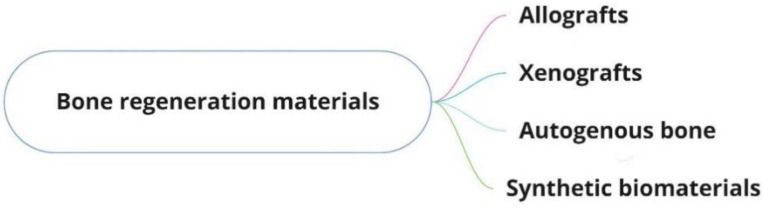
Flowchart of different bone regeneration biomaterials.

In cases of atrophy of the alveolar ridge or localized bone defects in the long term, peri-implant hard and soft tissues are disturbed. Alveolar resorption after tooth extraction occurs in the first year. Previous human studies have described horizontal bone loss of 29–63% and vertical bone loss of 11–22% during the first 6 months after tooth extraction. In addition, when the height of the alveolar ridge is more than 5 mm, procedures such as augmentation and implant placement can be performed simultaneously, as opposed to cases in which the height of the residual ridge is less than 5 mm and requires time for bone healing after biomaterial insertion and final implant placement. Nowadays, many different bone regeneration biomaterials such as allografts, xenografts, autogenous bone, and synthetic biomaterials are used to reduce dimensional changes of the alveolar ridge and stimulate bone regeneration [1,2,3]. The following flowchart shows the different biomaterials used in dental medicine for bone regeneration (Figure 1).

### 1.1. Allografts

The source of an allogeneic bone graft is an individual (i.e., a living donor or a cadaver) of the same species but of a different genotype. The advantages of this biomaterial are avoidance of a secondary surgical site and shortened procedure time. Some of the disadvantages of allogeneic grafts are infection, nerve damage at the donor site, and limited bone availability [4].

### 1.2. Xenografts

Xenografts are bone substitutes derived from animals such as cattle, pigs, and horses. Prior to use, such bone must undergo a mechanical and chemical purification process to remove organic components and eventually yield hydroxyapatite granules that closely resemble human bone. Xenografts are biocompatible and hydrophilic and have osteoconductive properties. Theoretically, bovine xenografts pose a risk of transmitting prion infections to the recipient, which is one of the disadvantages of this biomaterial. Research has shown that the risk of transmission of disease is negligible, but suspicion still exists. Xenografts are available in the form of bone blocks or granules (grafts made of small or large particles). Another disadvantage is that a xenogeneic bone block may fracture during fixation, affecting the surgical procedure and bone healing. Xenografts are used in the following cases: cavity preservation, sinus floor augmentation, and guided bone regeneration. In addition, due to their advantages in terms of mechanical properties and resorption resistance, they are often combined with autogenous bone to achieve volume stability [5,6,7,8].

### 1.3. Autogenous Bone

Autogenous bone is considered the gold standard for clinical bone augmentation. The material is completely biocompatible because the donor is the patient himself/herself. For this reason, an additional surgical site is needed from which the replacement is taken; this site can be intraoral or extraoral. One of the main problems with autogenous bone graft is resorption. The graft has a tendency to lose volume (40%) during healing and remodeling. Other shortcomings such as a different surgical site, limited availability, morbidity, risk of bleeding, edema, and postoperative pain have led to the development of new biomaterials [7,9,10,11,12,13,14,15].

### 1.4. Dentin Matrix

The first documented evidence of the osteoinductive potential of a demineralized dentin matrix was provided in 1967 by the detection of bone morphogenetic proteins (BMPs) in dentin [16]. Bone morphogenetic proteins belong to the TGF-β family and are the only signaling molecules that can independently induce de novo bone formation at orthotopic and heterotopic sites, and their presence in dentin primarily distinguishes them from xenogeneic biomaterials that do not contain proteins [11,17,18]. In 2003, dentin was first used clinically as an augmentation material in maxillary sinus augmentation [19]. Since 2008, dentin has been increasingly used as an augmentation material thanks to the development of devices that facilitate its clinical use [20].

In addition to BMPs, dentin contains both type I and type III collagen, as well as other growth factors, including insulin-like growth factor 2 (IGF-II) and transforming growth factor β (TGF-β) [21,22]. Most bone remodeling proteins such as osteopontin (OPN), osteocalcin (OCN), bone sialoprotein (BSP), osterix, type I collagen, and Cbfa1 (Runx2) have also been identified in dentin, making it an effective bone substitute [23,24,25,26].

### 1.5. Synthetic Biomaterials

Alloplastic bone grafts, which belong to the group of synthetic biomaterials, are used as an alternative to the gold standard. Advantages of these bone graft substitutes are their biocompatibility, osteoconductive capabilities, and stability. In addition, no donor site is required, and there is no risk of transmission of infectious diseases [21,22,23].

Synthetic bone substitutes represent a large group of inorganic biomaterials with different physical, chemical, and structural properties. Synthetic bone substitutes are composed of calcium phosphate to be as similar as possible to natural bone, which is mainly composed of calcium phosphate hydroxyapatite. The first experimental use of these biomaterials was reported in the 1920s [24]. Synthetic calcium phosphates include non-resorbable, rigid, friable hydroxyapatite (HA), resorbable β-tricalcium phosphate (β-TCP), and a complex called biphasic calcium phosphate (BCP) [9,24]. The HA does not resorb but acts as a scaffold to maintain space and integrity in the host bone defect, while β-TCP is fully resorbed, resulting in the stimulation of new bone through the release of calcium and phosphorus ions [24,25,26]. 

Synthetic biomaterials must be such that they do not cause inflammation and an inflammatory response. A proper balance between resorption of the scaffold and new bone formation is important for successful bone remodeling [9]. In addition, the integration of biomaterials and their degradation and vascularization may be influenced by the amount of cytokine and invasive inflammatory cell secretion. When tissue is damaged and the biomaterial is incorporated into the defect, inflammatory mediators are released from protein plasma and tissue, which adhere to the biomaterial. Such a cell layer leads to the integration of inflammatory mediators, of which macrophages should be highlighted, which are involved in the degradation and/or phagocytosis of the introduced biomaterial. In addition, depending on the size of the material the macrophages come into contact with, the overall cellular inflammatory response and granulation tissue formation are affected. Larger particles of size >500 µm with low porosity lead to better bone regeneration as they degrade more slowly than particles of size <50 µm. Therefore, in a study by Karabuda et al., in which three different biomaterials were used, the relationship between new bone formation and resorption of a particular biomaterial was observed. The results showed that the biomaterial with granules of size from 500 to 1000 µm contributed to a higher percentage of newly formed bone. In the same study, biomaterials with a smaller granule size were histologically found to have the most connective and marrow tissue after 6 months of healing. However, smaller particles allow filling of all defects and cannot prevent the ingrowth of connective tissue into the defect due to their rapid degradation. Therefore, according to Ghanaati et al., in purely synthetic biomaterials, incorporation of pure ß-TCP granules into the aqueous carrier system could prevent rapid degradation of biomaterials, especially in injection pastes where the granules are a bioactive filler and the aqueous phase contributes to material integrity as a carrier. Therefore, changes in the porosity, morphology, and particle size of a given biomaterial may affect the final result [3,27,28].

Various forms of CaP biomaterials exist on the market as powders, blocks, and granules in many sizes, which are difficult to handle, especially when applying bone material into three-dimensional cavities. These disadvantages have led to the development of materials in injectable form [21,29]. In addition, the increasing use of biomaterials in injectable form has become popular due to their viscosity and ease of use. This can lead to a better clinical outcome and a reduction in surgical time [21,30].

However, various clinical cases require the use of injectable bone substitutes (IBS) with certain additives. Most IBS are based on hydrophilic polymers such as collagen, hyaluronic acid (HY), and cellulose, in addition to calcium-phosphate-based granules. In a study by Barbeck et al., it was shown that the addition of HY and methylcellulose to β-TCP granules results in a biomaterial that plays an integrative role by inducing continuous cell growth from the periphery to the core, thus increasing vascularization around the implant [22]. In addition, authors of studies conducted on animal (Struillou et al. 2011) and human models (Weiss et al., 2007) support that the adjunction of silanized hydroxypropylmethylcellulose (Si-HPMC) interacts as a cohesive factor for BCP granules and contributes to better osteoconductive properties of the biomaterial and eventually to an excellent clinical outcome [31,32]. 

CaP cements without any additives usually show poor injectability due to liquid separation and a solid phase. In most cases, purely inorganic CaP pastes tend to disintegrate in the early stages of contact with biological fluids (blood) due to poor cohesion. Finally, the release of calcium phosphate particles into the bloodstream can cause certain complications; increased blood clotting can lead to disorders in the cardiovascular system causing, for example, pulmonary embolism. Numerous studies have been devoted to improving the aforementioned injection form of CaP cement by varying various factors such as composition, particle size, liquid-to-powder ratio, and processing during preparation. Moreover, many organic or inorganic additives such as citric acid, cytosan, gelatin, collagen, sodium alginate, polymer fibers, and their impurities are added to the powder or liquid phase to improve the handling and mechanical properties [33]. A parallel can be drawn to the study by Mai et al. (2012), conducted on an animal model, in which the combination of injectable calcium phosphate cement with polylactic co-glycolic acid (PLGA) improved the properties of the injectable biomaterial for the purpose of bone regeneration. In addition, in a study by Hoekstra et al. (2013), the addition of PLGA resulted in porosity, which increased the surface area of the CaP cement and ultimately led to direct contact of the biomaterial with the bone without soft-tissue intervention [17,34].

These studies show that even small amounts of certain additives can improve the injection properties and cohesion of CaP cement.

We can divide CaP cements into single-phase and two-phase cements. In general, single-phase CaP cements in injectable form are biocompatible and osteoconductive, but their degradation is generally slow. As noted in many animal studies, e.g., Guha et al. and Felix et al., the addition of polymeric microparticles is useful to increase the cement degradation rate. 

This is based on the fact that the degradation rate of sintered low-solubility cements can be significantly accelerated by introducing a secondary-phase CaP with higher solubility, such as ß-tricalcium phosphate (ß-TCP). Two-phase CaP cements consisting of α and ß components were shown to contribute to bone formation in a study by Jansen et al. Cements consisting of 85% α-TCP and 15% ß-TCP contributed to bone formation. Parallels can be drawn here with a study by Sariibrahimoglu et al., in which the two-phase nature of cements was compared. Two-phase CaP cement showed a better curing time and injectability compared to single-phase CaP cement. Further in vitro studies on this topic are needed to analyze the differences [29,35,36,37].

Thus, the main advantage of injectable forms of CaP cements compared to CaP cements in other forms is that they can be placed in the bone cavity by themselves without mechanical processing. This feature is important in clinical applications with various wider or narrower bone defects, which favors the further development of minimally invasive surgical procedures.

Knowing all this, alloplastic biomaterials and their design, i.e., use with syringes of various sizes, have become increasingly popular and are an ideal substitute for other types of biomaterials, with the ability to cover the borders of various defects in the oral cavity and, thus, increased osteoconductive properties. Animal and human studies on these injectable biomaterials play an important role in the field of dentistry [30,33,38].

The aim of this article was to systematically review the literature on the effects of injectable synthetic biomaterials and their advantages and disadvantages for new bone formation in the maxilla and mandible in animals and humans. 

## 2. Materials and Methods

A literature search was conducted in November 2020 via the National Medical Library, Washington, DC (MEDLINE PubMed), the Cohrane Central Register of Controlled Trials (Cohrane library), and a biomedical database (Embase) using the following terms: [dental biomaterials]. Terms such as (injectable) or (synthetic) or (alveolar bone regeneration) or (bone graft) or (sinus augmentation) or (extraction sockets) were added to exclude any off-topic research. In all, 501 articles were found. 

The inclusion criteria were:

- human studies;
- animal studies;
- English language studies;
- case reports, clinical cases, experimental pilot studies, randomized clinical trials, and preliminary studies;
- studies limited to the application of synthetic biomaterials in dentistry;
- studies limited to the injectable form of application;
- studies that included biopsy (histomorphological) and radiographic analysis; and
- studies that observed specific outcomes listed in Table 1 and Table 2.

The exclusion criteria were:

- studies that were not in English;
- studies that were performed on other bones (orthopedic surgery);
- studies that did not use synthetic biomaterial in injectable form; and
- in vitro studies.

## 3. Results

Animal studies show heterogeneity in terms of animal models, specific outcomes, follow-up time, and composition of the specific injectable biomaterial used. These characteristics are given in Table 1.

A detailed analysis of the individual studies revealed that most studies were conducted on dogs (seven), followed by studies on rats and sheep (two each) and mice and rabbits (one each). Different outcome variables were observed depending on the study: bone–implant contact (BIC), newly formed bone, and peri-implant bone density. The follow-up period also varied in these studies, usually ranging from 3 to 6 months after implantation of the biomaterial. The biomaterials used were in injectable form, composed of calcium phosphate cement (CPC) alone or with organic or inorganic additives, as shown in Table 1.

Heterogeneity was also demonstrated by human studies on maxillae and mandibles. In these studies, the following outcomes were observed: newly formed bone, connective tissue, residual injectable bone graft substitute, radiographic density, and residual bone height. The time points varied from 2 months to 3 years after implantation of the biomaterials, as shown in Table 2. The biomaterials used were similar in composition to those used in the animal studies, i.e., they consisted of calcium phosphate cement with organic or inorganic additives. 

**Figure 2:**
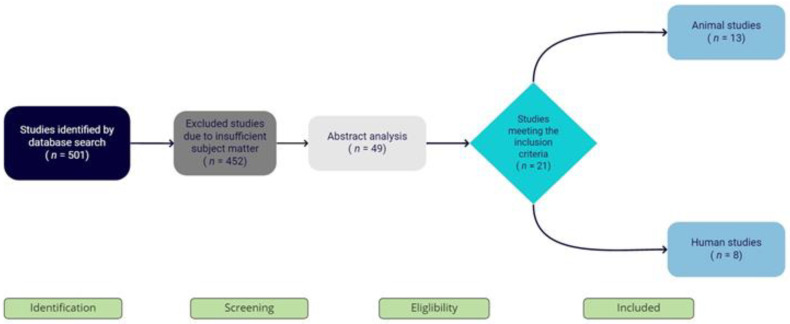
Prisma flowchart of search results.

Of the 501 articles screened, 452 were excluded due to insufficient subject matter. Abstract analysis was performed on 49 articles, resulting in 21 studies that met the inclusion criteria (13 animal and 8 human) (Figure 2).

We read the full text of the articles and classified them into studies that included research directly on maxillary or mandibular bone (16 studies) and research on periodontal tissue (5 studies). Little research has been conducted on this topic in the field of dentistry, and as far as we know, there is currently no study that provides a review of the literature on synthetic injectable biomaterials in dentistry in animals and humans. PICO criteria are shown in Table 3.

## 4. Discussion

The aim of this article was to systematically review the literature on the effect of injectable synthetic biomaterials and their advantages and disadvantages on new bone formation in the maxilla and mandible in animals and human.

### 4.1. Animal Studies

To our knowledge, Gauthier et al. (1999) [18] were among the first to perform animal studies on the use of injectable biomaterials and the ability of IBS to support new bone formation at fresh extraction sites in the maxilla and mandible after 3 months. The probing depth and bone ingrowth were greater in extraction sockets in the mandible than in the maxilla, which was not predicted from the literature [18]. At that time, the authors concluded that long-term studies would be useful to evaluate the biodegradation behavior of biomaterials, which leads us to conclude that 3 months after implant placement is a short period to observe biomaterial degradation. We also conclude that a material with injection properties and bioactivity supports new bone formation. The same authors found in a 2004 study on dogs that 3 months after healing, IBS triggers a significant increase in the bone–implant contact and peri-implant bone density compared with unfilled defects. The authors concluded that newly formed bone has the same Ca/P ratio with respect to basal bone [39]. This was also confirmed by the study by Aral et al. in 2008, in which injectable calcium phosphate cement was used. After 3 months, histological and histomorphometric analyses confirmed excellent bone biocompatibility and osteoconductive properties and no signs of inflammatory reaction, favoring new bone formation, comparable to autologous bone grafting [40]. However, one of the disadvantages of injectable biomaterials that should be considered during preparation is that the setting time must be long enough for the cement to mold into the defect. Furthermore, in 2011, Han et al. found that PRP from blood in combination with an injectable biomaterial can induce true bone regeneration as well as autogenous bone, because mesenchymal cells from peripheral blood have multidifferentiation potential [41]. These results indicate that proper preparation of a biomaterial with certain additives contributes to its mechanical strength and bioactivity. Further studies are needed on the use of human mesenchymal progenitor cells (BMPCs) and the possible use of steam cells for bone regeneration.

Most bone substitutes are composed of calcium phosphate to be as similar as possible to natural bone, which is mainly composed of calcium phosphate hydroxyapatite. Sa et al. (2017) found that the incorporation of millimeter-size, sintered HA particles significantly improves the osteoconductive behavior of porous injectable cement after 12 weeks in rabbits [42]. However, a recent 2020 study on mice by Kaneko et al. showed that the area with injectable biomaterial of higher viscosity and higher HA has a significantly lower percentage of newly formed bone [43]. However, as the authors noted, the disadvantages of these two studies are the relatively short time period and the small animal models. We can conclude that future research should focus on the biomechanical properties of biomaterials, such as cohesion during curing and the increase in viscosity, with different additives so that the injected form of the biomaterial meets all the criteria for minimally invasive surgical procedures.

Injectable biomaterials are also used in the regeneration of periodontal bone defects, as reported by Hayashi et al. (2006). Histometric analysis of periodontal tissue after 12 weeks showed new bone formation, new cementum, and new connective tissue attachment [44]. In this regard, Shirakata et al. (2012) confirmed that injectable graft material induces a high degree of new bone formation and cementum formation after 8 weeks of implantation [45]. The results of the 2012 study by Oortgiesen et al. showed that the addition of various organic or inorganic additives benefits the whole bone and periodontal ligament. The results of the same authors’ 2013 study on rats showed 50% more bone formation after 12 weeks in the group that received enamel matrix derivatives (EMD) added to the injectable biomaterial. The EMD/CaP combination has a synergistic effect, stimulating healing of soft periodontal tissue and bone regeneration [46,47]. 

Such results support previous publications in which combinations of injectable biomaterials with various additives have a synergistic effect, stimulating both soft periodontal tissue healing and bone regeneration, leading us to conclude that additives can increase the viscosity of the biomaterial, which, ultimately, when in the optimal viscosity range and reducing the separation phase, allows better handling and control by the therapist. Drawing conclusions across studies is difficult because studies differ in many aspects, such as differences in animal models (small defects), biomaterials used, and follow-up time. Nevertheless, some important speculations can be made. Biomaterials used in animal studies, alone or with certain additives, similar preparations in the phase-mixing process, and application techniques such as specially designed or ordinary syringes ultimately lead to easier handling and access to hard-to-reach sites, especially in small-animal models. This knowledge could lead to better clinical outcomes and a reduction in surgical time, as shown by the final results of the study in terms of newly formed bone, bone–implant contact, etc. 

### 4.2. Human Studies

Following the current literature on human studies in which injectable forms of biomaterials have been used, although few according to our search, the most relevant evidence in terms of successful regenerative potential and new bone formation is presented.

There are several human studies, the first of which were reported in 2004. Wolff et al. and Stanton et al. in the same year concluded that after different follow-up periods (2 months, 1 year, 3 years), injectable biomaterial was completely replaced by new bone and increased the height of the bone. Despite good clinical results, with no signs of infection and inflammation after so many years, no conclusion can be drawn from these two studies regarding the suitability of the material, because only a radiological examination was performed without histological analysis and a small amount of injected biomaterial was used for the majority of the patients [48,49]. Regarding the handling of biomaterials in these two studies, we can note that in the study by Stanton et al., there was leakage of the biomaterial from the defect, suggesting that the prepared biomaterial was too viscous and therefore the extrusion force during leakage was higher and the release of the biomaterial was difficult to control. Moreover, the final compressive strength of both biomaterials in these two studies (2.1 and 2.7 Mpa) was the same as that of cancellous bone. We can conclude that such newly formed bone is resistant to fracture, which is certainly a positive side of these biomaterials.

In recent studies by Papanchev et al. (2015) and Lorenz et al. (2018), at 4, 6, and 9 months after the augmentation procedure, histological and histomorphometric analyses of bone biopsy specimens showed a comparable amount of newly formed bone and connective tissue [21,50]. What stands out in the study by Lorenz et al. is that the biopsy samples were taken after a mean integration time of 4 months after the augmentation procedure, which is rather early compared to the recommendations for other bone graft substitutes. 

Moreover, we can conclude that some of the other biomechanical advantages of injectable biomaterials are their fluidity to fill three-dimensional cavities after tooth extraction. Therefore, solid biomaterials would be indicated in much more complex procedures such as sinus augmentation, where the biomaterial serves as a space maintainer. Comparing the studies of Weiss et al. and Lorenz et al. [21,32] in terms of biomaterial resorption, we conclude that the resorption process depends on the chemical structure of the biomaterial, i.e., particle diameter. Logically, the larger the particles of the biomaterial, the slower the resorption, which lead us to conclude that for procedures prior to implant placement in smaller extraction spaces, the use of smaller granules in injectable form, which are more rapidly resorbed and replaced by newly formed bone, leads to better clinical success. 

In addition, previous clinical and radiographic follow-up of implants placed in extraction sockets showed that IBS contributes to the long-term stability of the implants. Therefore, implants can be safely placed earlier, which is important to shorten the overall treatment time and provide comfort to the patient.

The studies by Khaled et al. and Georgiev et al. [51,52] using injectable biomaterials in combination with HA nanoparticles suggested that HA in the form of smaller granules contributes to better cellular interaction, leading to faster resorption of the biomaterial and promotion of new bone. We saw this ourselves when we used an injectable biomaterial with the addition of HA in a previous study [53]. Histomorphological and CBCT analyses showed that the granules integrate and are gradually replaced by newly formed bone. Such a result is consistent with the results of the two studies mentioned above.

Therefore, our analysis confirms that the use of injectable biomaterials is increasing in human studies. However, several critical issues arise from the results, namely the small sample size, different follow-up periods, the use of different biomaterials, and the exclusion of several studies due to the lack of quantitative analyses and biopsy collection sites.

Although this analysis is noteworthy, to the best of our knowledge, it is the first systematic review of biomaterials in injectable form used in animal and human studies.

## 5. Conclusions

The conclusions that can be drawn suggest that bone augmentation with injectable biomaterials increases bone volume and allows adequate implant placement in the atrophic maxilla and mandible. The injectable form of the biomaterial offers a modern way of insertion into the defect; more specifically, it can be adapted immediately after implant placement in three-dimensional defects and thus fits precisely into the defects, unlike other forms that are usually in the form of a block and need to be specifically adapted to each individual defect before insertion. Based on animal and human studies reviewed in this paper, the advantages of the injectable form of biomaterials are better handling and application to smaller defects in terms of delivery to hard-to-reach sites, reduction in surgical time, compressive strength, favorable tissue response, rapid resorption associated with the use of smaller particles with the formation of new bone, and the ability to mix the biomaterial with various additives that increase interaction between cells.

However, the disadvantages of injectable forms of biomaterials include the inability to use them in geometrically challenging large cavities that require the use of solid biomaterials due to larger granules, increased viscosity due to higher liquid content, and consequently more difficult injection and leakage. Due to all these points, further studies, especially in humans, based on histological and histomorphological analyses of biomaterials, with a better understanding of the biomechanical properties of injectable form delivery, are needed to draw more concrete conclusions that will contribute to a better understanding of the performance of this type of biomaterials and their role in alveolar bone regeneration.

## Figures and Tables

**Table 1:** Animal studies.

Author	Title	Year	Aim of the Study	Biomaterial	Preparation/Properties of Biomaterial	Animal Model	Outcomes	Follow-up Period (Months)	Reference
Mai et al.	Preliminary Application of Injectable Calcium Phosphate Cement/Poly(Lactic-co-Glycolic Acid) Microspheres for Extraction Site Preservation	2014	Assess the ability of injectable calcium phosphate cement (CPC) + poly(lactic-*co*-glycolic acid) (PLGA) microspheres.	Injectable calcium phosphate cement (CPC) + poly(lactic-*co*-glycolic acid) (PLGA) microspheres	No data	Dog	Newly formed bone 69.2% ± 1.8% 94.7% ± 1.1% 96.0% ± 0.9%	1 2 3	[17]
Gauthier et al.	A New Injectable Calcium Phosphate Biomaterial for Immediate Bone Filling of Extraction Sockets: A Preliminary Study in Dogs	1999	Assess the effects of IBS in bone regeneration.	Injectable CaP: BCP granules with 60/40 HA/β-TCP + cellulose polymer (MHPC 2%)	Application in ready-to-use glass flasks BCP granule diameter 200–500 µm	Dog	Newly formed bone: mandible (64.31% +/− 10.27%); maxilla (48.96% +/− 8.90%)	3	[18]
Struillou et al.	Treatment of Periodontal Defects in Dogs Using an Injectable Composite Hydrogel/Biphasic Calcium Phosphate	2011	Assess the ability of the hydrogel to promote the cohesion of BCP granules.	Hydrogel/BCP: BCP + Si-HPMC hydrogel; BCP granules (Biomatlante SARL, Vigneux de Bretagne, France)–hydroxypropyl methyl cellulose (HPMC, Colorcon-Dow Chemical, France)	BCP + Si: HPMC mixed in two sterile syringes BCP granule diameter 80–200 µm	Dog	Bone–material contact 61.3% ± 9.2% Bone ingrowth 35.5% ± 13.9%	3	[31]
Hoekstra et al.	Maxillary Sinus Floor Augmentation with Injectable Calcium Phosphate Cements: A Pre -clinical Study in Sheep	2013	Assess the biological performance of two types of injectable CPC: PLGA + PLGA microspheres.	CaP: 85% alpha-tricalcium phosphate (CAM Bioceramics BV, Leiden, the Netherlands) + 10% dicalcium phosphate anhydrous (Baker, Griesheim, Germany) + 5% precipitated hydroxyapatite (Merck, Darmstadt, Germany) PLGA (Purac Biomaterials BV, Gorinchem, the Netherlands) in two types: Purasorb^®^ PDLG 5002A and Purasorb^®^ PDLG 5002	Powder and liquid mixed in apparatus (Silamat) and shaken for 15 s PLGAL-AT microsphere size 37 ± 11 µm PLGAH-EC microsphere size 41 ± 10 µm	Sheep	Newly formed bone within the ROI CPC-PLGA_L-AT (low molecular weight)_ 26.4% ± 10.5% CPC-PLGA_H-EC (high molecular weight)_ 8.6% ± 3.9%	3	[34]
Boix et al.	Alveolar Bone Regeneration for Immediate Implant Placement Using an Injectable Bone Substitute: An Experimental Study in Dogs	2004	Quantitatively assess the different parameters of bone regeneration with IBS.	IBS: BCP granules with 60/40 HA/β-TCP + polymer cellulose derivative (MHPC) composite material obtaining by mixing 3% MHPC with BCP granules	Application in ready-to-use plastic injectors BCP granule diameter 40–80 µm	Dog	Terms of the number of threads in contact with bone 8.6% -bone–implant contact 11.0% -peri-implant bone density 14.7%	3	[39]
Aral et al.	Injectable Calcium Phosphate Cement as Graft Material for Maxillary Sinus Augmentation: An Experimental Pilot Study	2008	Assess the effectiveness of injectable CaP cement as a graft material.	Injectable calcium phosphate cement (Augmentech AT, Wetzlar, Germany)	Powder and liquid mixed in apparatus (Silamat) and shaken for 15 s Applicattion in ready-to-mix syringe system	Sheep	Bone–implant contact (BIC) 36% ± 5%	3	[40]
Han et al.	Alveolar immediate implants using around Immediate Implants Using an Injectable nHAC/CSH Loaded with Autogenic Blood -Acquired Mesenchymal Progenitor Cells: An Experimental Study in the Dog Mandible	2011	Assess new bone formation using nHAC/CSH + blood mesenchymal progenitor cells (dBMPC).	Injectable bone substitute powder composed of CSH and nHAC mixed with liquid	Application in a 5 mL syringe with a puncture needle	Dog	Bone–implant contact: dBMPC + nHAC/CSH 65.03% +/− 3.13% nHAC/CSH 33.13% +/− 7.29% Bone density: dBMPC + nHAC/CSH 61.74% +/− 3.6% nHAC/CSH 12.12% +/− 3.08%	3	[41]
Sa et al.	Bone Response to Porous poly(Methyl Methacrylate) Cement Loaded with Hydroxyapatite Particles in a Rabbit Mandibular Model	2017	Assess bone formation and the response to porous PMMA with or without (HA).	PMMA powder mixed with 1% dibenzoyl peroxide	Solid and liquid manually mixed HA particle size 0.5–1 mm	Rabbit	Bone ingrowth PMMA vs. PMMA-HA: no statistically significant difference PMMA vs. PMMA-HA: statistically significant difference in benefit of PMMA-HA	1 3	[42]
Kaneko et al.	Hydroxyapatite Nanoparticles as Injectable Bone Substitute Material in a Vertical Bone Augmentation Model	2020	Assess the benefit of bone graft gel containing hydroxyapatite nanoparticles.	Injectable bone substitute: two types of nano-HA gel (high, low viscosity) containing glycerin + carboxymethylcellulose matrix from SofSera (Tokyo, Japan)	Application in a syringe with a 25 G needle Average particle size 40 nm	Mice	New bone area significantly greater in the low-viscosity (35%) group than in the high-viscosity group (26%)	3	[43]
Hayashi et al.	Injectable Calcium Phosphate Bone Cement Provides Favorable Space and a Scaffold for Periodontal Regeneration in Dogs	2006	Assess the influence of injectable calcium phosphate bone cement.	Monocalcium phosphate monohydrate, α-tricalcium phosphate, and calcium carbonate + solution of sodium phosphate	Powder and liquid blended in a capsule for 20 s in amalgam mixer apparatus Application in specially designed applicator	Dog	New bone formation 4.90% ± 0.56%	3	[44]
Shirakata et al.	Effect of Bone Swaging with Calcium Phosphate Bone Cement on Periodontal Regeneration in Dogs	2012	Assess the effects of modified BS + CPC on periodontal healing.	Injectable CPC grafting materials (Norian PDC; Shofu Inc.,Kyoto, Japan)	Powder and liquid blended in a capsule for 20 s in amalgam mixer apparatus Application in specially designed applicator	Dog	Newly formed bone 3.53 ± 0.30 mm	2	[45]
Oortgiesen et al.	Periodontal Regeneration Using an Injectable Bone Cement Combined with BMP-2 or FGF-2	2012	Histologically assess the healing of CaP cement + BMP-2 or FGF-2.	Injectable CaP gel containing BMP-2 or FGF-2	Powder and liquid mixed in apparatus (Silamat) and shaken for 15 s Application in a 2 mL syringe	Rat	Bone formation: statistically significant difference in benefit of CaP/BMP group	3	[46]
Oortgiesen et al.	Regeneration of the Periodontium Using Enamel Matrix Derivative in Combination with an Injectable Bone Cement	2013	Histologically assess the healing of CaP + EMD.	Enamel matrix derivate (EMD); PLGA microparticles (Purasorb, Purac, Gorinchem, the Netherlands); CaP (85% alpha-tricalcium phosphate, 10% dicalcium phosphate, 5% precipitated hydroxyapatite)	Powder and liquid mixed in apparatus (Silamat) and shaken for 15 s Application in a 2 mL syringe PLGA microsphere diameter 26 +/− 8 µm	Rat	More bone formation EMD vs. CaP/EMD group (1.9 -> 2.9) 50%	3	[47]

**Table 2:** Human studies.

Author	Title	Year	Aim of the Study	Biomaterial	Preparation/Properties of Biomaterial	Outcomes	Follow-up Period (Months)	Reference
Lorenz et al.	Injectable Bone Substitute Material on the Basis of β-TCP and Hyaluronan Achieves Complete Bone Regeneration While Undergoing Nearly Complete Degradation	2018	Assess the regenerative potential and pathways of injectable bone substitute material.	Β-TCP mixed with an organic substance containing methylcellulose and sodium hyaluronate	Particles size < 63 mm	Newly formed bone 44.92% ± 5.16% Connective tissue 52.49% ± 6.43% Remnants of the IBS 2.59% ± 2.05%	4	[21]
Weiss et al.	The Safety and Efficacy of an Injectable Bone Substitute in Dental Sockets Demonstrated in a Human Clinical Trial	2007	Assess the safety of the filler material and the efficacy of the material for filling human tooth sockets and preventing bone loss.	Injectable calcium phosphate ceramic suspension (CAP ceramic particles suspended in a saline solution containing 2% hydroxylpropylmethyl cellulose)	Application in a 5 mL glass syringe BCP particle diameter 80–200 µm	Radiographic density of alveolar bone crest 76% ± 10% 84% ± 12%	3 6	[32]
Wolff et al.	Degradable Injectable Bone Cement in Maxillofacial Surgery: Indications and Clinical Experience in 27 Patients	2004	Treat the athrophic anterior mandible in combination with the insertion of dental implants.	Injectable CaP-Norian SRS (monocalcium phosphate monohydrate, tricalcium phosphate, calcium carbonate mixed with soidum phospahte solution)	Application of 5–10 mL of biomaterial ICPBC final compressive strength 2.1 MPA	- After 30 months, material completely replaced (only radiologically) - Height of the athropic mandible increased from 13 to 20 mm	12–30 (mean 29.2 months) -for patients who underwent enodsseous implants—11–26 (mean 15.5 months)	[48]
Stanton et al.	Injectable Calcium-Phosphate Bone Cement (Norian) for Reconstruction of a Large Mandibular Defect: A Case Report	2004	Reconstruct the large bone defect created by the enucleation of an odotogenic keratocyst.	Norian (Synthes Maxillofacial, West Chester, PA)	ICPBC final compressive strength 2.7 MPA	¼ of the Norian that was placed sequestered through the mucosa; progressive resorption of Norian and replacement with new bone (only radiologically)	2 36	[49]
Papanchev et al.	Comparison of the Rates of Bone Regeneration in Sinus Lift Grafting with Calcium Phosphate Paste between the 6th and 9th Month—A Clinical Case	2015	Find out whether there are significant differences in bone formation between the 6- and 9-month period after sinus lift grafting.	Maxresorb inject (Botiss Dental, Berlin, Germany)	Syringe	1. Operation: right sinus lift; 15% of newly formed bone 2. Operation: left sinus lift; 21% of newly formed bone	9 6	[50]
Khaled et al.	Maxillary Sinus Floor Elevation Using Hydroxyapatite Nanoparticles vs. Tenting Technique with Simultaneous Implant Placement: A Randomized Clinical Trial	2018	Assess the amount of bone height gain, density values, and implant stability after sinus augmentation with hydroxyapatite.	Nano-hydroxyapatite bone substitute (Nanostreams, HA nanoparticles (calcium phosphate nanoparticles, Nanostreams MC, Derby, Derbyshire, United Kingdom)	Disposabe syringe	- Bone height Nano group (7.0 ± 0.8 mm) Tent group (5.0 ± 1.5 mm) - Mean bone density Nano group (548 ± 25 HU) Tent group (420 ± 23 HU) - Mean ISQ value Nano group (78 ± 5) Tent group (77 ± 5)	6	[51]
Georgiev et al.	An Evaluation of Three-Dimensional Scans of the Time-Dependent Volume Changes in Bone Grafting Materials	2015	Compare volume loss between bone grafting materials.	Maxresorb inject (calcium phosphate paste composed of 80% nano-hydroxyapatite aquagel and 20% biphasic calcium phosphate granules)	Syringe	Bone graft volume loss 0.5256 cm³	36	[52]
Čandrlić et al.	Histological and Radiological Features of Four-Phase Injectable Synthetic Bone Graft in Guided Bone Regeneration—A Case Report	2021	Assess the efficacy of ISBG in the managment of bucal fenestration.	ISBG (Maxresorb inject, Botiss Biomaterials GmbH, Berlin, Germany) + native collagen membrane (Collprotect, Botiss Biomaterials GmbH, Berlin, Germany)	Syringe	ROI gray level 138.5 ROI gray level 454 Mineralized tissue 24.76% ISBG 12.56% Soft tissue 62.68%	10 days 6 months	[53]

**Table 3:** PICO criteria.

Patient and Population (P)	Human and Animal Studies
Intervention (I)	Application of injectable synthetic bone grafting materials in dentistry
Comparator or control group	Application of other types of bone grafting materials
Outcomes (O)	Newly formed bone, bone–implant contact, bone–material contact, bone ingrowth, bone density, remnants of IBS, connective tissue, radiographic density of alveolar bone crest, bone graft volume loss

## Data Availability

Data sharing is not applicable to this article.
